# Biostabilization of fecal sludge and tannery liming sludge: A novel approach

**DOI:** 10.1016/j.hazadv.2024.100500

**Published:** 2024-11

**Authors:** Md. Abul Hashem, Md. Enamul Hasan Zahin, Md. Anik Hasan, Mehedi Hasan, Tanvir Ahmed, Sk Shaker Ahamed, Md. Abu Hasan

**Affiliations:** a Department of Leather Engineering, Khulna University of Engineering & Technology (KUET), Khulna 9203, Bangladesh; b ITN-BUET Centre for Water Supply and Waste Management, Bangladesh University of Engineering and Technology (BUET), Dhaka 1000, Bangladesh; c Department of Civil Engineering, Bangladesh University of Engineering and Technology (BUET), Dhaka 1000, Bangladesh; d Fecal Sludge Management, Khulna City Corporation, Khulna, Bangladesh; e SAF Leather Industries Limited, Naopara, Jashore 7460, Bangladesh

**Keywords:** Fecal coliform, Helminth eggs, Salmonella spp., Germination index, Germination capacity

## Abstract

Fecal sludge and tannery liming sludge management is essential for humans and the environment. The emitted amount of waste from two industries is reduced in composting leading to value-added products. This research focused on the effectiveness and feasibility of co-composting fecal sludge and hair-burning liming sludge from tannery. Fecal sludge was intermittently mixed with hair-burning liming sludge which also consisted of chicken manure and sawdust. Five piles (Pile 1, Pile 2, Pil3, Pile 4, and Pile 5) indicated respectively as P#1, P#2, P#3, P#4, and P#5, contained various ratios of composting materials were mixed, piled onto a horizontal bamboo frame, and observed for 120 days. To ensure a suitable oxygen supply, the composting piles were turned on in the thermophilic stage so that pathogens could not survive. The highest temperatures in the thermophilic stage were 39.0°C, 49.2°C, 55.7°C, 41.7°C, and 51.3°C. These referred to the respective piles P#1, P#2, P#3, P#4, and P#5. The Dewar stability index confirmed the stability of each composting pile, and the maximum degradation was found for P#3. The metals chromium (Cr), zinc (Zn), lead (Pb), iron (Fe), and nickel (Ni) in the final compost were found to be 38.1, 144.7, 15.1, 450.5, and 22.7 mg/kg, respectively. TCLP results reveal only an insignificant amount of metal leaching. Fecal coliform of the compost was below the standard level; Helminth eggs and Salmonella spp. were absent. SEM micrographs reflect the decomposition of composting materials. The maximum germination index and germination capacity of compost pile P#3 for compost-soil ratio 1:0 and 1:1 were 90%-92.8% and 100%, respectively. The present approach produced nutrient-enriched compost from fecal sludge and hair-burning liming sludge from a tannery emerges as a suitable solution for reducing solid wastes.

## Highlights

• Biostabilization of fecal sludge and tannery liming for agricultural use.
• Produced nutrient-enriched compost's and metal level was within standard limits.
• Seed germination of the compost directs the aptness for soil conditioner.
• Bacteriological tests depict diminished Helminth eggs and Salmonella spp.
• TCLP indicates an insignificant amount of metal leaching from compost.

## Graphical abstract

**Figure:**
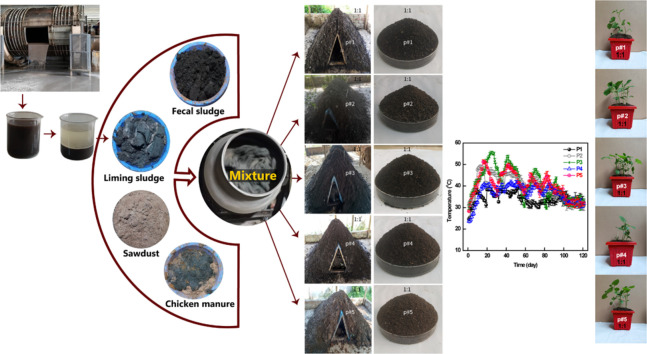

## 1 Introduction

Rapid urbanization and population growth result in increased fecal sludge generation every year. Globally, 80% of people have access to basic sanitation systems, but 20% of people are deprived of such facilities (Wanda et al., 2021). To get rid of human waste, 80% of people use the system of on-site sanitation, for instance, pit latrines and septic tanks. Even though these on-site systems accommodate a safe and private area to defecate, they are comparatively less effective in poor urban areas where streets are narrow and irregular, which makes it difficult for the suction truck to remove the fecal sludge (Onodera et al., 2014). It is challenging to estimate the pace at which fecal sludge builds up in septic tanks or latrines since it relies on a variety of factors, including groundwater level, tank size, number of users, and the time since the previous emptying (Nikiema et al., 2017).

Generally, when the septic tanks are full, the generated fecal sludge is culled from the system and typically disposed of directly in the agricultural fields or water bodies without treatment (Manga et al., 2021). This improper management affects the environment and endangers human health because it spreads water-borne diseases, which contain microorganisms, organic matter, and heavy metals (Wolf et al., 2019). Worldwide, one-third (1/3) of children below five years old are directly affected by water-borne diseases like dysentery, diarrheal, cholera, and typhoid (McGill et al., 2019; WHO, 2018). Furthermore, untreated fecal sludge is responsible for around 40% of the total greenhouse gas emissions. This has a serious effect on the environment and is a major contributor to climate change (Nsiah-Gyambibi et al., 2021).

Like fecal sludge, a large amount of liming sludge is generated from one of the most potential and economically important industries, the leather industry. In leather processing, several operations are involved, which ultimately produce a large amount of wastewater and sludge. It is calculated that from 1 Mg (Mega gram) of raw hide/skin, approximately 400–700 kg of solid waste and 15–50 Mg wastewater are generated (Chojnacka et al., 2021). Jiang et al. (2016) added that the amount of sludge generated by the leather industry is almost equal to 60% of the total weight of the raw hide/skin. Liming is an essential step in leather processing in a tannery, resulting in the production of around 60-70% of the total tannery sludge (Hasan et al., 2022). In Bangladesh, some 40,800 tons of sludge are generated yearly from the liming process alone. This sludge is unfortunately discarded haphazardly without being treated in any way (Payel et al., 2021). Because sludge contains several organic components, heavy metals, and other dangerous substances, improper or negligent management of it poses a serious environmental as well as public health risk (Zhai et al., 2020).

The predominant techniques for managing tannery sludge are landfilling and anaerobic digestion. However, there is a significant likelihood of generating secondary pollutants through both technologies (Liu et al., 2019). Hence, proper management of fecal sludge and liming sludge, taking into account the environmental, economic, and human health context, has become indispensable. However, both fecal sludge and liming sludge contain an abundant amount of organic compounds and nutrients, which, if not utilized properly or efficiently, can seriously impact the environment. Nartey et al. (2021) and Hashem et al. (2021) asserted that significant quantities of vital elements such as nitrogen (N), phosphorous (P), potassium (K), and carbon (C) are found in both fecal sludge and liming sludge, which are crucial for promoting plant growth. Therefore, organic fertilizer could be produced from the fecal sludge in combination with liming sludge. The calcium and sulfur content in liming sludge might assist in soil fertility (Hashem et al., 2021). A combination of fecal sludge and liming sludge would increase the organic substance that might facilitate microbial degradation, which will reduce the large amount of waste from two sources.

Mostly organic composts are used to increase soil fertility with sufficient nutrients for plant growth (Ayilara et al., 2020). It is beneficial in pollution prevention (Uyizeye et al., 2019), weed control (Coelho et al., 2019), bioremediation (Ventorino et al., 2019), and so on. Latifah et al. (2019) stated the composting mechanism into four stages based on temperature dynamics. Temperature ranging from 10-50°C, microbial activity rises 2-3 times/10°C (Nozhevnikova et al., 2019). Microbial degradation of the organic matter in wastes accelerates during the thermophilic stages and the active microbes absorb the persistent organic pollutants as well (Semple et al., 2001). The temperature gradually comes back to the mesophilic stage when most of the organic matter is being digested and finally comes to the ambient temperature (Zamri et al., 2021). Sawdust accelerates the degradation activity by elevating the temperature profile (Huang et al., 2006). Chicken manure was imparted as a nitrogen source to the process (Li et al., 2022). The land-filling generates leachate as a secondary pollutant but in aerobic composting is less than land-filling (Ozkaya, 2005). In aerobic composting, pathogens present in organic wastes are mostly eliminated with high temperatures due to microbial metabolism (Kokhia, 2015).

An investigation was done of the co-composting of fecal sludge in combination with the tannery hair-burning liming sludge containing chicken manure and sawdust. This study examined the physicochemical parameters at different times and spacings during aerobic composting. The bacteriological (Fecal coliform, Helminth eggs, and Salmonella spp.), nutrient, and SEM analyses of the produced compost were undertaken to assess environmental compatibility. The heavy metal in the final compost, leaching behavior, and their correlation were examined. The germination of the seed and the growth of the plant in the compost were examined.

## 2 Materials and methods

### 2.1 Material collection

*In situ*, limed wastewater collection as well as limed sludge formation were executed at SAF Leather Industries in Jashore, Bangladesh. Immediately following the completion of the liming process, the effluent wastewater was gathered in a plastic drum. The gradual addition of aluminum sulfate (Al_2_(SO_4_)_3_) to the liming wastewater was completed by maintaining a pH level of 8.5-9.0 to inhibit the formation of hydrogen sulfide (Dixit et al., 2015). After settling for 6 h, the liming sludge was collected in a burlap sack and brought back to the waste treatment plant of Khulna University of Engineering & Technology (KUET), Khulna, Bangladesh. The fecal sludge was collected from the Fecal Sludge Treatment Plant (FSTP) at Khulna, Bangladesh. The raw fecal sludge was emptied from the tanker to the screening chamber to remove all grit and debris. Fecal sludge from the chamber was directed to the respective unplanted drying bed. After two weeks of drying, the dried fecal sludge was collected for co-composting with tannery liming sludge. The other associated composting materials e.g. sawdust, and chicken manure, were procured from local industries and farms at Khulna, Bangladesh.

### 2.2 Reagents and chemicals

For chemical and biochemical measurements, analytical-grade reagents were used. All reagents and commercial grade aluminum sulfate were purchased from a store in Khulna, Bangladesh.

### 2.3 Experimental model

This composting experiment was conducted under aerobic conditions. The composting materials were piled onto the triangular horizontal bamboo frames with dimensions L × W × H = (180 × 160 × 75) cm^3^. These frames were constructed for five piles and indicated P#1, P#2, P#3, P#4, and P#5, respectively.

### 2.4 Compost piling

**Fig. 1:**
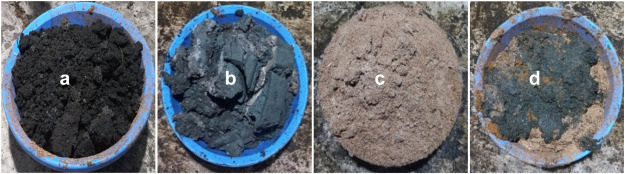
Composting materials (a) fecal sludge (b) liming sludge (c) sawdust and (d) chicken manure.

Fecal sludge, tannery liming sludge, sawdust, and chicken manure were mixed at different proportions for each pile to a definite weight of 600 kg. Pile 1 was set aside as the control for comparison. The initial moisture contents of fecal sludge (FS), liming sludge (LS), sawdust (SD), and chicken manure (CM) were found to be 45.0%, 41.9%, 40.4%, and 38.7%, respectively, and subsequently adjusted with the weights. Table 1 depicts the various proportions of each pile. Water was sprinkled to maintain moisture at 50-60% which is favorable for bacterial growth (Cofie et al., 2009). The composting process was observed for 120 days. Fig. 1 represents the composting materials. The FS was blackish, LS was dark greenish, the SD was a natural brown color and the CM was greenish brown.

**Fig. 2:**
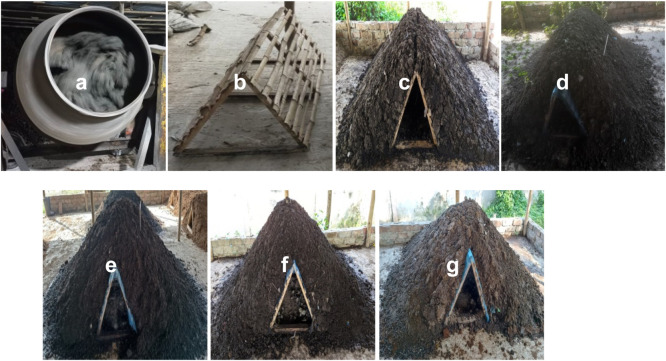
Compost materials mixing machine (a) horizontal bamboo frame (b) composting pile 1 only fecal sludge (c) composting pile 2 fecal sludge and liming sludge (d) composting pile 3 fecal sludge, liming sludge, and sawdust (e) composting pile 4 fecal sludge, liming sludge and chicken manure (f) and composting pile 5 fecal sludge, liming sludge, sawdust, and chicken manure (g).

The composting materials were mixed with the mixing machine intermittently and the mixed materials were piled up manually on the horizontal bamboo frame. The composting experiment was conducted under a tin shade environment to avoid direct sunlight. To monitor the piles’ temperatures, a thermometer was inserted at different places of these piles. While recording the temperature, the thermometer was inserted at different places to establish the mean values and standard deviation. Meanwhile, the daytime temperature was recorded. Fig. 2 reflects the mixing machine, horizontal bamboo frame, and composting piles.

### 2.5 Investigation of compost quality

#### 2.5.1 Temperature profile and turning frequency

The temperature profile is one of the key monitoring factors that guide compost maturation. A mesophilic reaction (∼25-40°C) commences in the early stages of composting, which rises to above 40°C (>40°C) when a thermophilic reaction occurs (Mihelcic, 2018). The daily temperature of each pile was recorded from every possible place of the piles that remained in an open condition. While the temperature started declining, turning off the pile was conducted manually to ensure aeration (Tiquia et al., 2002). The turning phase was continued during the week as part of the thermophilic stage and once every two weeks after finishing the thermophilic stage. The piles were mixed thoroughly after each turning cycle. Moreover, to observe any anaerobic change in the compost, the same mixing composting materials were inserted into the thermo flask. For monitoring the regular anaerobic temperature, a thermometer was inserted on the top of the thermo flask by drilling and sealed with glue to make it airtight. This insulated reactor system provided the biological stability index and self-heating activity based on the recommendation made by BGK (1994). Compost stability was confirmed from this self-heating temperature for 72 h following the Dewar test TMECC 05-08-D method. A retention time of 120 days was taken for experiments.

#### 2.5.2 Physicochemical properties of compost

Different parameters of the compost- pH, moisture content, electrical conductivity (EC), salinity, total dissolved solids (TDS), dry matter (DM), total solids (TS), fixed solids (FS), and volatile solids (VS) were determined. Following EPA 9045D (2004), a sample of 5±0.1 g was agitated in DI water of 100 mL for 24 h and run through a pH meter (BT-675, BOECO, Germany). Similarly, the EC and salinity of the filtrate were evaluated through a conductivity meter (CT-676, BOECO, Germany). The TDS content of the compost was measured by using the APHA 2540D standard procedure. ISO 8190: 1992’s loss on ignition (LOI) technique was applied to detect the moisture content. A sample of 5 g was dried for 5 h at 103±2°C in an oven. Any evident mass difference between the samples before and after dehydration was computed. For determining TS, FS, and VS, a sample of 25±0.1g was oven-dried at 103±2°C for 12 h following EPA 1684.

Residues obtained from this were cooled and ignited again at 550°C for 2 h. The mass differences before and after drying were calculated to determine the TS, FS, and VS, respectively. BS EN 12879 (2000) and the DM (%) and TDS content of the compost were measured following APHA 2540D guidelines. The presence of metals- chromium (Cr), zinc (Zn), lead (Pb), copper (Cu), iron (Fe), Ni (nickel), and lead (Pb) of the acid-digested aliquot of the final compost was determined through atomic absorption spectroscopy (Spectra240FS AA, Agilent, USA) following EPA 3050B standard. Triplicate data were taken for each element. The obtained outcomes were compared with Bangladesh's standard of organic fertilizers (Islam, 2006).

#### 2.5.3 NPKS analysis

After being oven-dried for 24 h at 103±2°C, the compost was then pulverized to 2 mm sieving size for the assessment. The Total Kjeldahl Nitrogen (TKN) method was carried out following AOAC (1995) for the nitrogen content assessment. The molybdovanadate method of spectrophotometer was conducted for phosphorous (P) determination (Estrin and Brammell, 1969). Potassium (K) and sulfur (S) content were determined by flame photometer and turbidimetric methods, respectively. Elemental relation was identified through Pearson's correlation coefficient following equation (i).

(1) $$r=\frac{\sum \left(x_{i}-\bar{x}\right)\times \left(y_{i}-\bar{y}\right)}{\sqrt{\sum {\left(x_{i}-\bar{x}\right)}^{2}}\times \sqrt{\sum {\left(y_{i}-\bar{y}\right)}^{2}}}$$

Where ‘r’ is the co-efficient of correlation, ‘x_i_’ denotes the 1st variable, ‘$\bar{x}$’ stands for the average of the 1st variables, ‘y_i_’ represents the 2nd variable, and ‘$\bar{y}$’ is the average of the 2nd variables. Pearson's variable value ranging from 0.3-0.7 reflects the strongest correlation between the two variables.

#### 2.5.4 Analysis of compost morphology

SEM images of the final compost at 50 KX magnifications with 15 kV were analyzed (JEOL JSM-6490, USA), the purpose being to observe the compost's surface morphology.

#### 2.5.5 Maturity and seed germination assessment

Compost maturity and seed germination were determined by taking 100% compost from each pile in five crocks and a 50:50 compost soil ratio in another five crocks, respectively. Gourd (*Lagenaria siceraria*), pumpkin (*Cucurbita moschata*), and okra (*Abelmoschus esculentus*) seeds were planted in each crock and left for a 12-day incubation period. Sufficient water was sprinkled during this incubation period. Later on, a few plants were uprooted to determine the root length, and one plant was allowed to grow for investigation of its physiological properties- plant height, and plant weight. Germination index (GI) and germination capacity (GC) were determined using the following equations (ii) and (iii):

(2) $$\text{GI}(\%)=\frac{\text{Relative}\;\text{germination}\;\text{of}\;\text{seeds}}{\text{Relative}\;\text{germination}\;\text{of}\;\text{roots}}\times 100$$

(3) $$GC\left(\%\right)=\frac{Germinated\;seed\;No.}{Total\;seed\;No.}\times 100$$

#### 2.5.6 Elemental analysis

The matured compost was pulverized and sieved through a mesh screen of 2 mm for elemental analysis after being dried for 24 h at 103±2°C in an oven. The oven-dried sample amount of 1-2 g was put into a digestion vessel with 9 mL HNO_3_ and 1 mL H_2_O_2_. The microwave digester was turned on at 220°C for 20 min. After cooling, the digested solution was filtered, and elements were quantified through AAS.

#### 2.5.7 TCLP test

To evaluate the mobility of elements contained in the matured compost, the Toxicity Characteristic Leaching Procedure (TCLP) test was carried out following the US EPA 1311 (1992) standard. Any liquid fraction of TCLP extracts that exhibits levels of controlled heavy metals at such high concentrations, as determined by analysis, is deemed hazardous waste. The concentration will remain higher than the permitted limit for certain metals even after a considerable dilution from the other extract fractions. For the TCLP test, 25 g dried compost (sieved to 9.5 mm) was taken into a plastic cylinder where 0.57% (v/v) acetic acid was poured at a constant liquid-to-solid ratio (20:1). A pH of 2.81±0.05 was found in the combination. The AAS assessed the metal concentration after the cylinder was shaken for 18 h at 30±2 rpm and a 0.45 µm filter paper was used to filter the leachate.

### 2.6 Bacteriological analysis

#### 2.6.1 Fecal coliform

The amount of fecal coliform (FC) was analyzed following the Most Probable Number (MPN) method (APHA 9221E, 2005). A sterile laboratory mixer was used to combine 450 mL of distilled water with a 50 mL compost sample homogeneously that was obtained from four dilutions of the sample. The sample was then serially diluted at different dilution factors. A 1 mL sample was taken from each diluted sample into three tubes, each containing 10 mL of prepared A-1 broth media. Subsequently, the tubes were conditioned at 35±0.5°C for 3 h and at 44.5±0.2°C for 24 h for incubation. Turbidity and inner tube gas production were assessed to measure the presence of FC. The amount of FC was determined by applying the formula as follows:

FC (MPN/100 mL) = Chart MPN × sample for the 1st column of the chart (mL)/sample in the 1st dilution of the selected series (mL).

#### 2.6.2 Helminth eggs

The microscopy method (based on the UKZN PRG Helminth Method) was followed to analyze the Helminth eggs of the final compost. The compost sample was first preserved at 4-10°C, providing adequate moisture. A 10-20 g compost sample was taken in a 200 mL beaker with 50-80 mL AmBic and stirred for 10 min. The mixture was sieved through a mesh of 100 µm to 20 µm, which acted as a filter. The bottom sieve was monitored for fluid generation after washing the 100 µm mesh out completely. The 20 µm sieves were separated, rinsed out with distilled water, and taken into a test tube. The tube was then centrifuged for 10 min at 3000 rpm. The obtained sediment was taken, mixed with 3 mL zinc sulfate (ZnSO_4_), and centrifuged for 10 min at 2000 rpm. The obtained supernatant again passed through the mesh of 100 µm to 20 µm and collected the fluid of 20 µm mesh. The fluid's centrifugation at 3000 rpm was conducted for 10 min. Then the sediment was placed on microscope slides to observe Helminth egg numbers calculated per liter of the sample.

#### 2.6.3 Salmonella spp

The Most Probable Number (MPN) method (adopted from Modified Semisolid Rappaport-Vassiliadis (MSRV) medium, US EPA: Method:1682) served to detect Salmonella counting where culture media was prepared to cultivate the species. This test includes the enrichment phase, selection phase, and biochemical confirmation phase. A compost sample of 30±0.1g was put into a sterile blender along with 270 mL DI water and mixed homogeneously. The sample was brought to a pH of 7.0–7.5. The sample was diluted by 10^-4^ times. The diluted sample in the tube was incubated at 36±1.5°C for 24±2 h. Six 30 µL drops of the sample were dispersed into six different segments of MSRV medium, which later on were placed into an incubator at 42±0.5°C for 17±1 h. The edge of the halos formed within those six regions was taken further for XLD (Xylose-lysine desoxycholate agar) analysis. One XLD plate was incubated at 36±1.5°C for 21±3 h, while another plate was stored at 1-5°C for back-up analysis. For the biochemical confirmation phase, the XLD colony was put into three inoculating tubes, TSI (Triple sugar iron agar), Urease, and LIA (Lysine iron agar). Each one had an inoculation for 24±2 h at 36±1.5°C and was tested for polyvalent positive or negative growth. The amount of Salmonella spp. was determined through equation (iv):

(4) $$\text{MPN}/4g=\frac{\left(\text{MPN}/\text{mL}\;\text{wet}\;\text{weight}\;\text{of}\;\text{the}\;\text{sample}\right)\times 4}{\%\;\text{of}\;\text{solids}\;\text{in}\;\text{decimal}}$$

## 3 Results and discussion

### 3.1 Physicochemical properties of composting materials

**Fig. 3:**
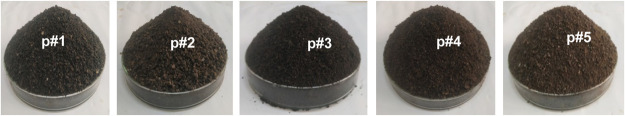
Matured compost of each pile (P#1, P#2, P#3, P#4, and P#5).

To facilitate the composting, the recommended pH is between 6.5 and 8.5. Karnchanawong et al. (2017) recommended neutral pH for microbial activity regulation. Higher pH initiates the volatilization of ammonia, which creates a bad odor (Hung et al., 2014). Table 2 depicts the dry matter (%), moisture content (%), and pH of raw composting materials. The pH, dry matter, and moisture content of raw composting materials were 11.4–5.8, 61.3%–59.6%, and 38.7%–45.0%, respectively. The physicochemical properties of the mixed raw composting ingredients and matured compost are shown in Table 3 and Table 4, respectively. Fig. 3 depicts the matured compost. The pH of mixed raw composting ingredients for P#1, P#2, P#3, P#4, and P#5 was recorded as 5.5, 5.6, 6.1, 6.0, and 6.2, respectively. In the case of matured compost, the pH levels were 6.4, 6.5, 6.6, 6.3, and 6.5, respectively.

During sludge formation, aluminum sulfate (coagulant) was added into the liming wastewater to maintain pH 8.5-9.0. The rest alkali in liming sludge might neutralize through mixtures of other components that could facilitate the degradation of organic matter to generate nutrients during composting. A pH of around 8.0 is suggested in composting (Wong et al., 2017). However, the pH of the raw composting ingredients’ mixture may be lowered by the organic acid generated during the initial phase of decomposition. These organic acids are, later on, converted into methane and carbon dioxide and increased the pH (Guo et al., 2020). Discrete sources of nutrient content and bulkiness of the ingredients guided towards variable volatile solids content. The breakdown of composting materials through microbes reduces the amount of volatile solids (Hill and Baldwin, 2012). The amount of VS of the final compost for P#1, P#2, P#3, P#4, and P#5 was obtained at 53.8%, 48.3%, 44.7%, 44.6%, and 46.6%, respectively. Microbes break down the composting materials in the presence of air by emitting odor gases therefore; VS was reduced (Zhang et al., 2013). In aerobic composting, VS emits carbon dioxide, in the case of anaerobic composting, it produces biogas (e.g. methane).

Similarly, the breakdown of organic matter increases the salinity, TDS, and EC of the final compost piles. Hashem et al. (2023) observed similar results during composting of tannery limed fleshing. Salinity and EC associated with nutrient ions help in plant growth, reduce ion-toxicity, and facilitate the soil's water absorption efficiency (Goswami et al., 2017; Shrivastava and Kumar, 2015). The carbon-nitrogen ratio of the final compost recorded less than the initial amount, which also indicates the decay of organic content. For compost piles P#1, P#2, P#3, P#4, and P#5, the carbon-nitrogen ratios were 24.7, 23.5, 25.5, 24.2, and 26.9, respectively. Al-Khadher et al. (2021) recommended the carbon-nitrogen ratio of compost <20 after maturation. The final characteristics of the compost parameters satisfied the standards set by Switzerland (Fuchs et al., 2001), Great Britain (PAS 100, 2005), and Indian standards (BIS 16556, 2016), respectively, so the maintenance of compost inherent quality was certified.

### 3.2 Temperature profile

**Fig. 4:**
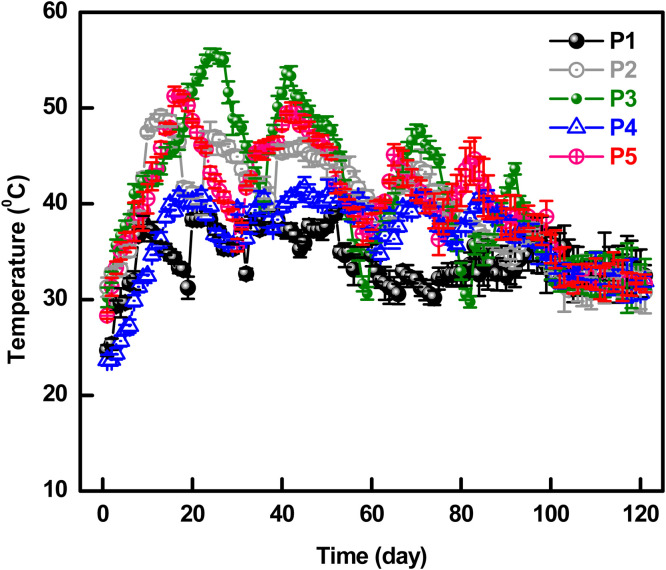
Temperature profile of the compost piles for 120 days.

Temperature plays a vital role in confirming the maturity of compost (Wang et al., 2019). Fig. 4 depicts the gradual increase of temperature for each pile, which indicates the breakdown of proteins, fats, and carbohydrates through thermophilic bacteria within 40°C. Salama et al. (2016) recommended that 50-55°C is the best for maximum decay of pathogenic bacteria. Turning off the composting piles was continued to ensure sufficient aeration necessary for the functioning and quick growth of these bacteria. The scarcity of oxygen might produce a bad odor for anaerobic bacterial growth. Pathogenic bacteria are disabled and tend to decay at this high temperature (Nakasaki and Hirai, 2017). Maximum temperatures of the aerobically set piles were recorded at 39.0°C, 49.2°C, 55.7°C, 41.7°C, and 51.3°C for P#1, P#2, P#3, P#4, and P#5, respectively. The temperature for P#1 (on the 52nd day), P#2 (on the 13th day), P#3 (on the 24th day), P#4 (on the 52nd day), and P#5 (on the 16th day) remained above 40°C up to a certain period. Temperature was decreased when the rate of decay was completed.

**Fig. 5:**
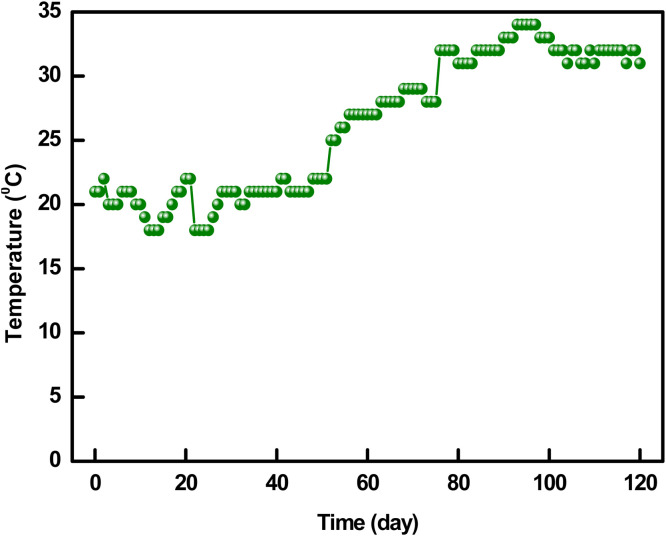
Day temperature profile of the compost during 120 days.

This period of the thermophilic stage was enough for the total elimination of the microorganisms for each pile (Vico et al., 2018). P#3 showed the maximum temperature (55.7°C) and remained the highest thermophilic period (up to the 93^rd^ day) to degrade the composting materials. The remaining auxiliary ingredients were decomposed, afterward leading to a decline in metabolic activity but elevating the nitrification rate (Bernal et al., 2009). Fig. 5 depicts the day temperature required to observe the temperature rise, which ranged between 18 and 34°C within the whole composting period (120 days). The temperature was recorded at 21°C on the 1st day of composting; during the process, the lowest temperature was recorded at 18°C on the 13th day and the highest was 34°C on the 94th day. The pile temperature was found to be unaffected by the day temperature. On the 104th day, day temperature was recorded as 32°C while P#1, P#2, P#3, P#4, and P#5 temperatures were 33.7°C, 31.0°C, 32.7°C, 32.7°C and 32.6°C, respectively.

### 3.3 Stability of the compost

**Fig. 6:**
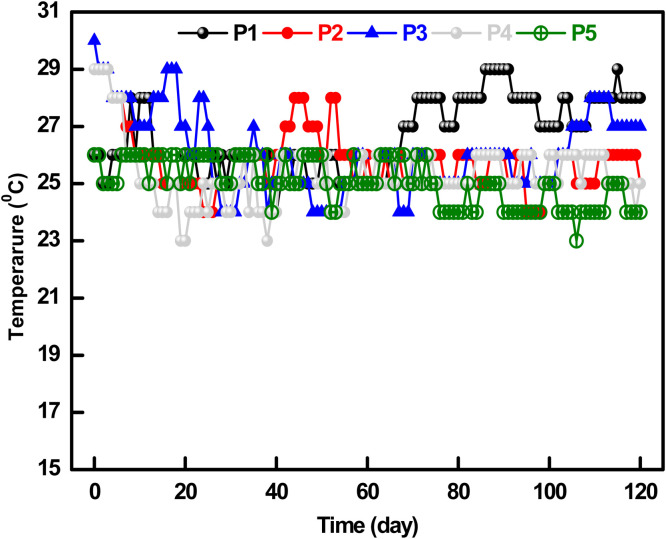
Self-heating temperature profile of the compost piles.

Fig. 6 depicts the compost stability curve of the self-heating temperature monitored in a thermoflask during composting to ensure anaerobic conditions. The temperature for P#1, P#2, P#3, P#4, and P#5 was recorded as elevated from day temperature till the 54th day after it was declined. Decomposing of organic materials might elevate the temperature and declination of this temperature indicates the compost maturation. The highest temperature value, T_max_ (°C), the highest increase of temperature per hour, I_max_ (°C/h), and the area underneath the curve after 72 h, A_72_ (°Ch) were investigated for each pile following Dewar test TMECC 05-08-D method. Researchers suggest the effectiveness of the self-heating test method as a stability indicator (Butler et al., 2001; Koenig and Bari, 2000). This test method suggested for a <10°C temperature rise “Very stable (>V),” for a 10-20°C temperature rise “Stable (V),” and for a >20°C temperature rise “Unstable (<V)” rating, respectively, as represented in Table 5. All the piles revealed a similar pattern of stability index indicating compost maturation (Wichuk and McCartney, 2013) and P#3 inhibited the maximum degradation rate of organic matter (A_72_=7320°Ch) compared to P#1 (A_72_=6360°Ch), P#2 (A_72_=7308°Ch), P#4 (A_72_=7308°Ch), and P#5 (A_72_=6552°Ch).

### 3.4 Compost quality

#### 3.4.1 NPKS

Compost refers to an enriched nutrient source provided with macro-elements, which are evident and beneficial for plant growth. A few proteolytic bacteria transform N-NO_3_ and N-NH_4_ into organic acids, amide, and aliphatic nitrogenous form. The humic compounds in fecal sludge and tannery liming sludge break down through microorganisms in aerobic conditions resulting in nutrient-rich compost (Ayilara et al., 2020; Shah et al., 2021; Amir et al., 2004).

Table 6 depicts the nutrient content of the matured compost. The N, P, K, and S of the compost ranged from 1.15-3.55%, 0.31-0.75%, 0.29-54%, and 1.65-3.65%, respectively. It seems that the N content for all piles is within the Bangladesh standard (0.5-4.0%). The lowest and the highest N content were found at 1.15% and 3.60% in P#1 (only FS) and P#2 (FS and LS), respectively. The maximum N was detected in P#2 (3.60%). Hence, except P#1, N content in other compost piles was the highest range of Bangladesh standard (4.0%). The highest and the lowest P were evident in P#3 (0.75%) and P#1 (0.31%). P content beyond the below level was in P#1 (0.31%) and P#5 (0.40%). Likewise, the highest K content was observed in P#3 (0.54%), while in the remaining piles, K was below the lower level of the Bangladesh standard. The S content of all piles was above the highest limit of the Bangladesh standard (0.5%). Except for S content, P#3 compost fulfilled the requirements of Bangladesh standards.

#### 3.4.2 Bacteriological analysis

Table 7 depicts all the reduction of pathogens satisfying the EPA 503 limit for all the piles except for P#1 (only fecal sludge). In pile P#1, FC was 1100 MPN/g, which exceeds the standard limit (<1000 MPN/g). In the case of compost piles P#2, P#3, P#4, and P#5, FC content amounted to 430, 36, 74, and 30 MPN/g, respectively. Except for pile P#1, the pile temperature rose during composting. During composting, continuous biological heating led to the thermal inactivation of several pathogens (Wiley and Westerberg, 1969). According to the EPA 503 rule, the hygiene premise states that the amount of FC bacteria serves as an indication of the existence of Salmonella, commonly found in sludge (US EPA, 1998; Yanko, 1988). For compost made from Class A biosolids, the EPA established a maximum limit of 1000 MPN/g for fecal coliform (FC); compost levels over 2 × 10^6^ MPN/g were classified as Class B material for restricted application (US EPA, 1998). Ascaris eggs are rendered inactive by exposure to temperatures exceeding 45°C for a minimum of 5 days. Elevated temperatures accelerate the rate at which Ascaris cells dry up and impair their ability to retard desiccation as the total Helminth eggs (Kone et al., 2007).

#### 3.4.3 Heavy metal analysis

Heavy metals play a major role in fulfilling the ion deficit in soil, which facilitates plant growth but only in limited amounts (Mikula et al., 2020). An abundance of heavy metals in the matured compost indicates the phenomena of leaching out, which adversely affects the soil and water (Chen et al., 2020). Table 8 depicts the heavy metals to be present in the compost piles. The amounts of Cr, Zn, Pb, Fe, and Ni were found to be 13.5-38.1, 121.6-144.7, 7.3-15.1, 376.7-450.5, and 12.1-22.7 mg/kg, respectively. All the metals seemed to be in the satisfactory range as reported by Enayetullah et al. (2006). Hashem et al. (2023) also noted similar ranges of heavy metals in the tannery limed-fleshing compost. Inter-elemental correlation is depicted in Table 9. This relationship suggests that macro-elements have the strongest association between P-N, K-N, K-P, S-P, and S-K (coefficient values of 0.3-0.7).

Among micro-elements, Cr bonded strongly with all the macro-elements, including Cr-N, Cr-P, Cr-K, and Cr-S (coefficient values of 0.18–0.69). Among the macro-nutrients, the strongest (+ve) correlation existed between Cr-P, Zn-Cr, Pb-Cr, Cu-P, Fe-Pb, and Ni-Cu. Similarly, the strongest (-ve) correlation was found between Zn-S, Pb-Zn, Cu-K, Fe-K, and Ni-N. All the remaining correlation values reflected moderate to strong correlation except for the Cu-S correlation. This type of significant correlation was documented in previous studies, and these macro and micro-elements have a good effect on plant growth (Hannet et al., 2021).

#### 3.4.4 Leaching behavior of metals

Table 10 depicts the leaching of metals from the produced compost. The compost was evaluated using TCLP to observe whether it is suitable for placement on land or should be disposed of as hazardous waste. The metal found in each compost pile was within the US EPA permissible limit except Zn in P#1 (only FS). While associate materials, namely tannery liming sludge, chicken manure, and sawdust were added at various ratios, the leached amount of Zn was below the permissible level. The degradation of organic matter causes metal formation, which is encapsulated through natural minerals. The alkaline liming sludge might reduce the organometallic complex formation by neutralizing through organic acids (Singh and Kalamdhad, 2012). The formation of metal complexes might result in a negligible amount of leaching. Chiang et al. (2007) stated that the rate of metal leaching reduces when the duration of composting is increased. Since the composting was observed for 120 days to mature, the leached amount of metal was small.

#### 3.4.5 Seed germination capacity

**Fig. 7:**
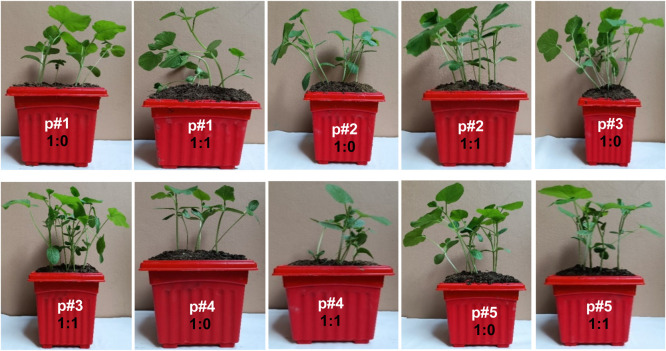
Seed germination capacity of the matured compost piles in different compost and soil ratios following P#1 (C:S=1:0), P#1 (C:S=1:1), P#2 (C:S=1:0 P#2 (C:S=1:1), P#3 (C:S=1:0), P#3 (C:S=1:1), P#4 (C:S=1:0), P#4 (C:S=1:1), P#5 (C:S=1:0), and P#5 (C:S=1:1).

The illustration in Fig. 7 highlights the observed plant growth, which serves as an indicator of the compost's germination capacity. It is plausible that an increase in pH and EC levels could potentially hinder biological processes and impede the growth of plants (Meng et al., 2019). Cesaro et al. (2019) suggest that the delayed germination of seeds in the soil can be attributed to the presence of phenols, alkaloids, ketones, and flavonoid compounds. It was observed for the development of gourd, pumpkin, and okra plants, which indicates the mature compost contains sufficient nutrients and biomass activity (Eudoxie and Alexander, 2011). According to Negi et al. (2020), compost is considered immature if its GI value is below 80%. The physiological properties of the plants cultivated in ten crocks for compost-soil ratio reported the following: P#1 (1:0; 1:1), P#2 (1:0; 1:1), P#3 (1:0: 1:1), P#4 (1:0; 1:1), and P#5 (1:0; 1:1), a high degree of similarity was exhibited. Table 11 provides a comprehensive overview of the height of the plant weight, which was remarkably consistent across all the compost ratios. The GI for every plant for all ratios as well as all composts showed matured germination properties. P#3 showed a maximum GI (90% to 92.8%) for both compost-soil ratios of (1:0; 1:1), where the GC was 100%. The seed germination experiment suggests the potential utilization of the produced compost as a viable fertilizer for agricultural purposes.

#### 3.4.6 SEM analysis

**Fig. 8:**
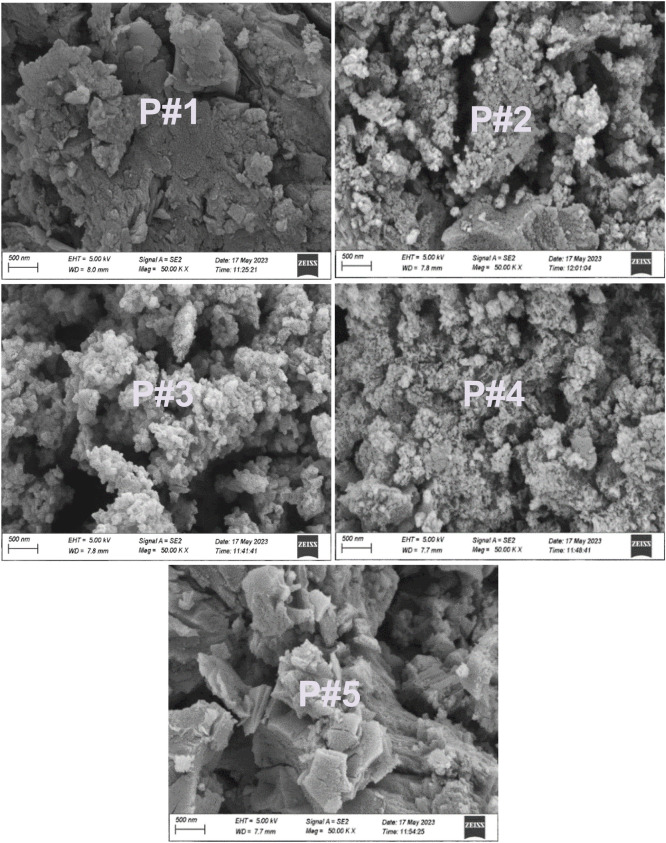
SEM analysis of final compost P#1, P#2, P#3, P#4, and P#5.

Fig. 8 depicts the SEM photographs of the final compost. The aggregation of biomass is caused by the individual breakdown and formation of new bondings between the matrices (Soobhany, 2018). This reveals the microbial decay of the non-fibrous proteins from each element to produce proper compost. A similar pattern was illustrated during composting through tannery-limed fleshing (Hashem et al., 2023).

## 4 Conclusion

This study investigated the degradation of fecal sludge in combination with tannery liming sludge. The physicochemical properties of the matured compost were recorded as satisfactory. The nutrient (NPKS) content of the compost (Pile 3) in combination with fecal sludge, liming sludge, and sawdust fulfilled the Bangladeshi standard except for sulfur. The temperature profile in composting indicates the organic matter degradation and destruction of fecal coliform, Helminth eggs, and Salmonella spp. The SEM micrographs confirmed the disintegration of the fecal sludge and liming sludge into well-matured compost. Seed germination revealed the potential application of the compost produced can applied to agricultural activities. The combination of liming sludge and fecal sludge composting offers a novel approach to turning municipal and tannery solid waste into recycled and valuable resources.

## Statement and declarations

The authors declare that the submitted manuscript is original. Authors also acknowledge that the current research has been conducted ethically and the final shape of the research has been agreed by all authors. The authors declared that this manuscript does not involve researching about humans or animals.

## Availability of data and materials

The datasets used and/or analyzed during the current study are available from the corresponding author upon reasonable request.

## CRediT authorship contribution statement

**Md. Abul Hashem:** Writing – original draft, Supervision, Project administration, Methodology, Investigation, Funding acquisition, Formal analysis, Conceptualization. **Md. Enamul Hasan Zahin:** Writing – review & editing, Visualization, Methodology, Investigation, Formal analysis, Data curation. **Md. Anik Hasan:** Investigation. **Mehedi Hasan:** Investigation. **Tanvir Ahmed:** Writing – review & editing, Funding acquisition. **Sk Shaker Ahamed:** Investigation. **Md. Abu Hasan:** Investigation.

## Declaration of competing interest

All authors have participated in (a) conception and design, or analysis and interpretation of the data; (b) drafting the article or revising it critically for important intellectual content; and (c) approval of the final version.

This manuscript has not been submitted to, nor is under review at, another journal or other publishing venue.

The authors have no affiliation with any organization with a direct or indirect financial interest in the subject matter discussed in the manuscript.

## Data Availability

The datasets used and/or analyzed during the current study are available from the corresponding author upon reasonable request.
